# Evaluating Paratransgenesis as a Potential Control Strategy for African Trypanosomiasis

**DOI:** 10.1371/journal.pntd.0002374

**Published:** 2013-08-15

**Authors:** Jan Medlock, Katherine E. Atkins, David N. Thomas, Serap Aksoy, Alison P. Galvani

**Affiliations:** 1 Department of Biological Sciences, Oregon State University, Corvallis, Oregon, United States of America; 2 Yale School of Public Health, New Haven, Connecticut, United States of America; IRD/CIRDES, Burkina Faso

## Abstract

Genetic-modification strategies are currently being developed to reduce the transmission of vector-borne diseases, including African trypanosomiasis. For tsetse, the vector of African trypanosomiasis, a paratransgenic strategy is being considered: this approach involves modification of the commensal symbiotic bacteria *Sodalis* to express trypanosome-resistance-conferring products. Modified *Sodalis* can then be driven into the tsetse population by cytoplasmic incompatibility (CI) from *Wolbachia* bacteria. To evaluate the effectiveness of this paratransgenic strategy in controlling African trypanosomiasis, we developed a three-species mathematical model of trypanosomiasis transmission among tsetse, humans, and animal reservoir hosts. Using empirical estimates of CI parameters, we found that paratransgenic tsetse have the potential to eliminate trypanosomiasis, provided that any extra mortality caused by *Wolbachia* colonization is low, that the paratransgene is effective at protecting against trypanosome transmission, and that the target tsetse species comprises a large majority of the tsetse population in the release location.

## Author Summary

African sleeping sickness is a fatal disease occurring in sub-Saharan Africa. The parasites that cause African sleeping sickness are transmitted between humans and livestock by the tsetse fly. Controlling the spread of the parasite by tsetse flies has been proposed as a promising strategy for reducing the incidence of sleeping sickness. One potential control method relies on releasing genetically modified tsetse that are resistant to carrying the sleeping sickness parasite. For this strategy to be successful, resistant tsetse must be able to invade the susceptible tsetse population. Here, we used a mathematical model to assess the feasibility of such a strategy and the implications for sleeping sickness prevalence in humans and livestock. We found that the strategy has the potential to eliminate sleeping sickness, provided that the genetic modification is effective at protecting against trypanosome transmission and provided that the target tsetse species comprises a large majority of the tsetse population in the release location.

## Introduction

African trypanosomiasis infects 30,000 people in sub-Saharan Africa. Without treatment, infection is almost always fatal [1]. In addition, losses in livestock production due to African trypanosomiasis are estimated at US$1 billion annually [2]. Currently, control efforts primarily target the tsetse vector by insect traps, insecticide spraying of land and livestock, and sterile-insect technique [3].

Transgenesis of infectious-disease vectors is being widely considered as a possible strategy for controlling the burden of vector-borne disease [4]–[7]. For tsetse, instead of trypanosomiasis-refractory genes being incorporated into the tsetse genome directly, these genes may be encoded in *Sodalis*, commensal bacteria that colonize the gut of tsetse [8]. This mode of gene expression is termed paratransgenesis, where changes in the insect are induced by modifying genes of a commensal organism.

*Wolbachia*, bacteria that are transmitted by female insects to their progeny, could be harnessed to drive pathogen-refractory transgenes into insect vector populations [9], [10]. Some strains of *Wolbachia* cause cytoplasmic incompatibility (CI), where the sperm of *Wolbachia*-colonized males is less competent than the sperm of non-colonized males at fertilizing non-colonized eggs [11]. Because of CI, when *Wolbachia* is at high frequency in the population, *Wolbachia*-colonized females have higher reproductive success than non-colonized females because mates are likely to be colonized males. At low frequency, *Wolbachia*-colonized females may have a fitness disadvantage if *Wolbachia* reduces egg count or increases mortality. Thus, a threshold in *Wolbachia* frequency may exist above which *Wolbachia* increases tsetse fitness and is therefore driven to fixation [9], as has been observed in natural and laboratory populations [12], [13]. Moreover, tsetse laboratory lines and some wild populations are naturally colonized by CI-inducing *Wolbachia* [14], [15], suggesting the feasibility of using *Wolbachia* as a gene driver. *Wolbachia*-induced CI gives paratransgenesis a powerful potential advantage to over existing vector-control methods: CI could prevent trypanosomiasis-tolerant tsetse from re-invading treated areas and could allow paratransgenic tsetse to invade and replace neighboring populations.

We evaluated the effectiveness of a paratransgenic strategy, transgenic *Sodalis* driven by *Wolbachia*, to control African trypanosomiasis. To do this, we developed a mathematical model combining the population genetics of CI-inducing *Wolbachia* in tsetse with the transmission dynamics of *Trypanosoma brucei gambiense*, the parasite responsible for 95% of reported human cases [1], among tsetse, humans, and non-human animal reservoir hosts. Previous mathematical models have examined the invasion of *Wolbachia* into populations of fruit flies [9], mosquitoes [16], and tsetse [17], but the analysis we present here is the first to incorporate the epidemiology of the trypanosome disease system with the population genetics of CI dynamics. Parametrizing this model with empirical estimates of CI, we assess the potential effectiveness of a trypanosome-refractory paratransgenic intervention to control trypanosomiasis.

## Methods

To understand the dynamics of *Wolbachia* colonization in a tsetse population, we combined a model of CI with a dynamic transmission model for trypanosome infection. We used this model to evaluate the effectiveness of a paratransgenic tsetse release on trypanosomiasis by concurrently tracking the coupled dynamics of *Wolbachia*, tsetse, humans and animal reservoir hosts in trypanosome endemic areas in Africa. We outline the model construction here; see Text S1 for more detail.

### *Wolbachia* dynamics in tsetse

Cytoplasmic incompatibility has been modeled using discrete-time models, with both non-overlapping generations [18]–[21] and overlapping generations [16], [22]. We extended our previous continuous-time model of *Wolbachia*–tsetse dynamics [17], based on reproductive rates of tsetse mating pairs (Table 1), to include age structure in the tsetse population (Text S1). Tsetse were divided into 10-day age classes [23] and mating was assumed to occur in the first adult age class [24]. This age-structured tsetse model enabled us to examine the effects of tsetse remating by allowing a proportion of both paratransgenic, *Wolbachia*-colonized and wild-type, non-colonized female tsetse to mate a second time [25]. We explicitly tracked the *Wolbachia* status of mating partners. For a given tsetse mating pair, reduction in reproductive rate was reduced by four parameters: first, the proportion of nonviable zygotes of non-colonized eggs by *Wolbachia*-colonized sperm, 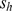 (e.g. 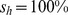 indicates that all incompatible fertilizations fail, which is perfect CI); second, the fecundity benefit of *Wolbachia*-colonized females relative to non-colonized females, 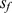 (e.g. 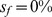 implies there is no fecundity benefit associated with *Wolbachia* colonization); third, the proportion of *Wolbachia*-colonization females having non-colonized offspring, termed 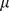 (e.g. 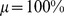 implies perfect transmission from females to their offspring); and last, an increased mortality rate for *Wolbachia*-colonized tsetse, 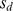 (e.g. 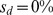 implies no extra mortality cost). In our empirical scenario we set 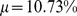, 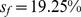, 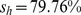, and 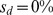 [17], but varied these parameters in the sensitivity analysis. We assumed that the paratransgenic tsetse are all released at one time into the target area, not continually released over several years. We also assumed that the paratransgenic tsetse released were newly emerged adults with a sex ratio equal to that of the wild population.

**Table 1:** Reproductive success of tsetse–*Wolbachia* crosses.

		Female			
		*Wol*−		*Wol*+	
**Male**	***Wol−***	*Wol*−	1	*Wol*−	*μ*(1+*s_f_*)
				*Wol*+	(1−*μ*)(1+*s_f_*)
	***Wol+***	*Wol*−	1−*s_h_*	*Wol*−	*μ*(1+*s_f_*)(1−*s_h_*)
				*Wol*+	(1−*μ*)(1+*s_f_*)

Shown are the relative reproductive rates resulting from tsetse–*Wolbachia* mating crosses, relative to the cross between a non-colonized female and a non-colonized male. All viable offspring are listed as a result of each mating cross. Parameters governing the reproductive rates of each mating are: 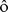, the relative egg-count increase due to *Wolbachia* colonization; 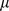, the proportion of eggs of *Wolbachia*-colonized females that are non-colonized; and 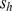, the proportion of fertilizations of colonized eggs by non-colonized sperm that are not viable due to CI [9]. Non-colonized females can only produce non-colonized offspring, whereas colonized females can produce both colonized and non-colonized offspring.

### Trypanosomiasis dynamics in tsetse, humans, and animals

**Figure 1:**
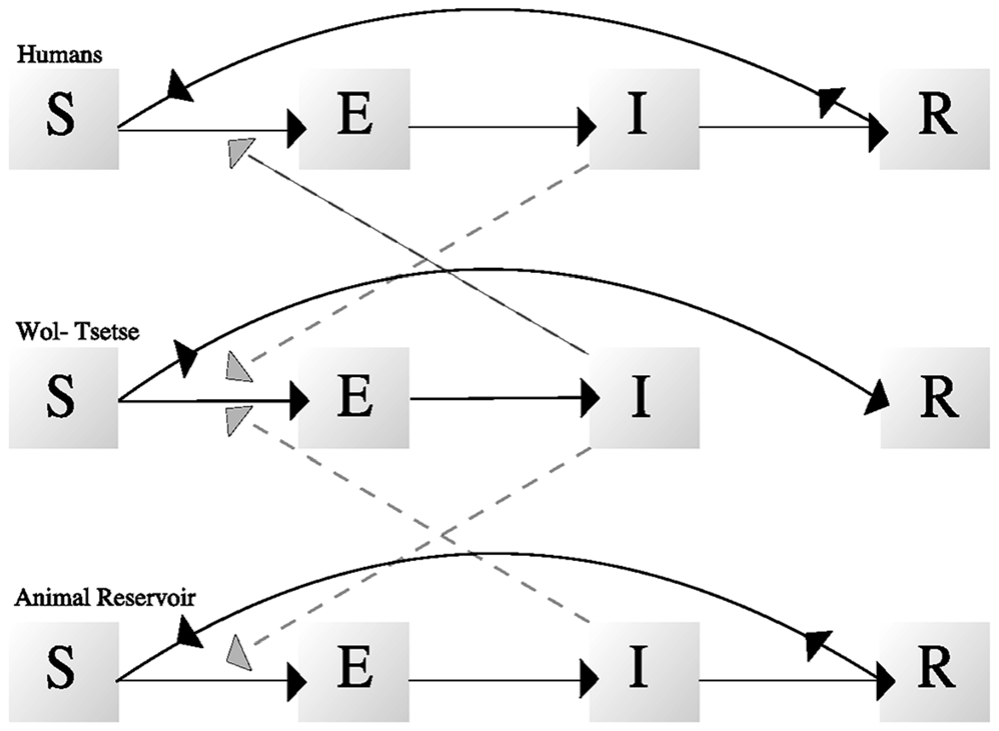
Trypanosomiasis transmission between species and progression of infection within individuals. Boxes 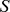, 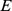, 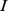, and 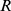 represent susceptible, exposed, infected, and recovered states, respectively. Solid black curves represent all possible state changes for an individual of a given species, and dashed gray curves represent the transmission of trypanosomes. Humans and animals are infected with trypanosomes based on the proportion of tsetse infected, biting rates, and transmission probabilities. Humans and animals then move through the exposed and infected states, and enter the recovered state after clearance, where they are both immune and non-infectious. After acquired immunity wanes, humans and animals return to the susceptible state. Tsetse that are not colonized by *Wolbachia* can become infected during their first blood-meal as a function of biting rates, proportions of infected hosts, and transmission probabilities. Tsetse that become infected move into the exposed state, and then the infected state, where they remain for life. Tsetse that are not infected with trypanosomes during their first blood-meal move into the recovered state, as they are no longer at risk of infection with *T. brucei*. Tsetse colonized by paratransgene-driving *Wolbachia* are assumed to be born immune to trypanosome infection and remain so for life: they have been omitted from this diagram.

We constructed a three-species SEIR differential-equation model for *T. b. gambiense* trypanosome infection among tsetse, humans, and animal reservoir hosts based on a previously published model [26]. Tsetse were modeled by combining our age-structured model for *Wolbachia* with a dynamic model of trypanosome infection (Figure 1 and Text S1).

Our model assumptions are consistent with the modeling study of Rogers [26], unless otherwise specified (Table 2). We assumed that tsetse are only susceptible to trypanosome infection during their first blood-meal and only within 24 hours after emergence from pupa to adult. Should susceptible tsetse become infected after feeding on an infectious vertebrate, the tsetse enter the exposed state during which the trypanosome infection incubates. After incubation, tsetse enter the infectious state and can transmit infection to humans and the animal reservoir. We further assumed that tsetse do not clear trypanosomes. Susceptible tsetse that do not become infected enter the resistant state after their first blood-meal or 24 hours after emergence, whichever comes first.

**Table 2:** Empirical model parameter values.

Parameter		Value	Reference
*H*	Human population size	300	[26]
*L*	Animal reservoir population size	50	[26]
*K_V_*	Tsetse population scaling constant	16538	[26]
*ε_V_*	Relative reproductive cost to tsetse of tryp. infection	0%	[39]
*a_H_*	Biting rate on humans	0.075 per day	[26], [40]
*a_L_*	Biting rate on animals	0.0175 per day	[26], [40]
*θ_c_*	Proportion of female tsetse with incompatible sperm that mate twice	0%	[30]
*θ_i_*	Proportion of female tsetse with compatible sperm that mate twice	0%	[30]
*m*	Tsetse birth rate	0.05 per day	[23], [26]
*ρ*	Tsetse adult survival rate	0.975 per day	[23], [26]
*ρ_P_*	Tsetse pupal survival rate	0.9946 per day	[23]
*β_VH_*	Human-to-tsetse transmission rate	0.065 per day	[26]
*β_VL_*	Animal-to-tsetse transmission rate	0.065 per day	[26]
*β_H_*	Tsetse-to-human transmission rate	0.62 per day	[26]
*β_L_*	Tsetse-to-animal transmission rate	0.62 per day	[26]
1/*τ_V_*	Tryp. incubation time in tsetse	25 days	[26]
1/*τ_H_*	Tryp. incubation time in humans	12 days	[26]
1/*τ_L_*	Tryp. incubation time in animals	12 days	[26]
1/*λ_H_*	Duration of tryp. infection in humans	70 days	[26]
1/*λ_L_*	Duration of tryp. infection in animals	50 days	[26]
1/*δ_H_*	Tryp. immune period in humans	50 days	[26]
1/*δ_L_*	Tryp. immune period in animals	50 days	[26]
*μ*	Proportion of eggs not *Wol*. colonized from *Wol*.-colonized females	10.73%	[17]
*s_h_*	Proportion of non-viable fertilizations due to CI	79.76%	[17]
*s_f_*	Relative increase in egg count due to *Wol*. colonization	19.25%	[9], [17]
*s_d_*	Relative increase in mortality rate due to *Wol*. colonization	0%	[9]

The dynamics of humans and animal reservoir trypanosomiasis infection each follow a standard vector-transmission model, in which individuals are divided into susceptible, exposed, infectious, and recovered states (Figure 1). Susceptible vertebrate hosts may become exposed after being bitten by an infectious tsetse. After incubating the infection, exposed vertebrates enter an infectious state in which they are capable of transmission to tsetse. Vertebrate hosts clear the infection and enter a recovered state in which they are immune to re-infection. After this immunity wanes, vertebrates return to being susceptible to trypanosomiasis. Both human and animal populations are of constant size with no births or deaths. Our base-case model assumed that paratransgenic tsetse are completely resistant to trypanosomiasis, that there is no tsetse remating, and that only one tsetse species inhabits the intervention location. We explore the sensitivity of results to these three assumptions.

## Results

**Figure 2:**
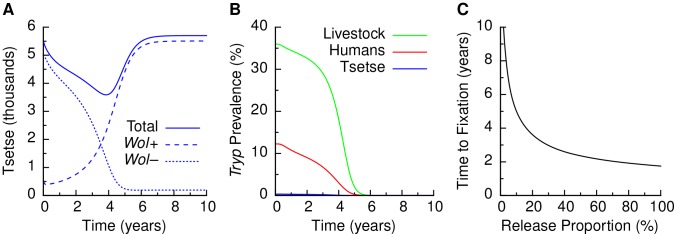
Model behavior following release of paratransgenic tsetse. (A) Tsetse population dynamics after a release of 20% paratransgenic *Wolbachia*-colonized tsetse enter the wild-type non-colonized tsetse. (B) Trypanosomiasis prevalence in host populations corresponding to the tsetse release shown in A. (C) Time to 95% *Wolbachia* fixation. The size of the *Wolbachia* paratransgenic tsetse released is given as a percent of the wild type tsetse population size. A larger release yields a shorter time for paratransgene fixation. Empirical parameter values (Table 2) are used in all results.

We used empirical estimates from laboratory experiments on the tsetse *Glossina morsitans morsitans* [17] for our base-case values of the CI parameters: transmission failure (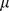), the inviability of fertilizations of *Wolbachia*-colonized eggs by sperm from non-colonized males (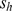), and fecundity benefit of *Wolbachia* colonization (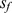). Mortality cost of *Wolbachia* colonization (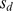) has not been estimated empirically for tsetse, so we assumed 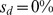 for the base case [9] and varied it in sensitivity analyses. For these empirical parameter values, we found that a paratransgene release can achieve the elimination of trypanosomiasis among humans, the animal reservoir, and tsetse (Figure 2A & B).

We also found that the abundance of paratransgenic tsetse released determines the speed at which trypanosomiasis elimination is achieved. Specifically, the larger the tsetse release, the faster the fixation of the paratransgene occurs in the tsetse population, but with diminishing returns as release abundances increase (Figure 2C). For example, for a paratransgenic release that is 10% of the abundance of the wild tsetse population, the paratransgene reaches 95% frequency in 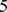 years, compared to 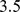 years for a release abundance of 20%. Moreover, *Wolbachia* establishment eradicates trypanosome infections in humans, animal reservoir, and tsetse at approximately the same rate as *Wolbachia* fixation (Figure 2A& B).

The dynamics of fixation are characterized by an initial decrease in the abundance of tsetse due to CI, followed by a recovery of the tsetse population as *Wolbachia* invades (Figure 2A). *Wolbachia* affects the equilibrium tsetse abundance by its fecundity benefit, by its mortality cost, and by the incompatibility between non-colonized eggs from colonized females and sperm from the almost completely *Wolbachia*-colonized population of males. For the empirical parameter values, the fecundity benefit (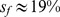) is sufficient to more than offset transmission failure (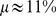) and mortality cost (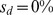), resulting in a post-fixation population that is about 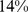 larger than the wild-type population, but the size of which also depends on the mechanisms that determine the carrying capacity.

### CI parameters

Trypanosomiasis is transmitted by multiple tsetse species [24], between which *Wolbachia*-CI parameter values may vary. Therefore, we calculated the effect of varying the CI parameters on the effectiveness of a paratransgenic release. Depending on the parameters, the model can exhibit a threshold for the size of the paratransgene release such that if the abundance of paratransgenic tsetse released are below this threshold, paratransgenic tsetse are driven out of the population, whereas if the release is larger than the threshold, paratransgenic tsetse are driven to fixation. These dynamics arise because *Wolbachia*-induced CI generates a frequency-dependent fitness effect: at low frequencies, the incompatibility of *Wolbachia*-colonized females with the predominantly non-colonized males imposes a fitness cost to colonization, while at high frequencies, the incompatibility of non-colonized males with the predominantly colonized females confers a fitness advantage to the colonized tsetse. This frequency-dependent fitness is in addition to the frequency-independent effects of the fecundity benefit (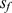) and mortality cost (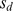) of *Wolbachia* colonization. (See also Text S1.)

**Figure 3:**
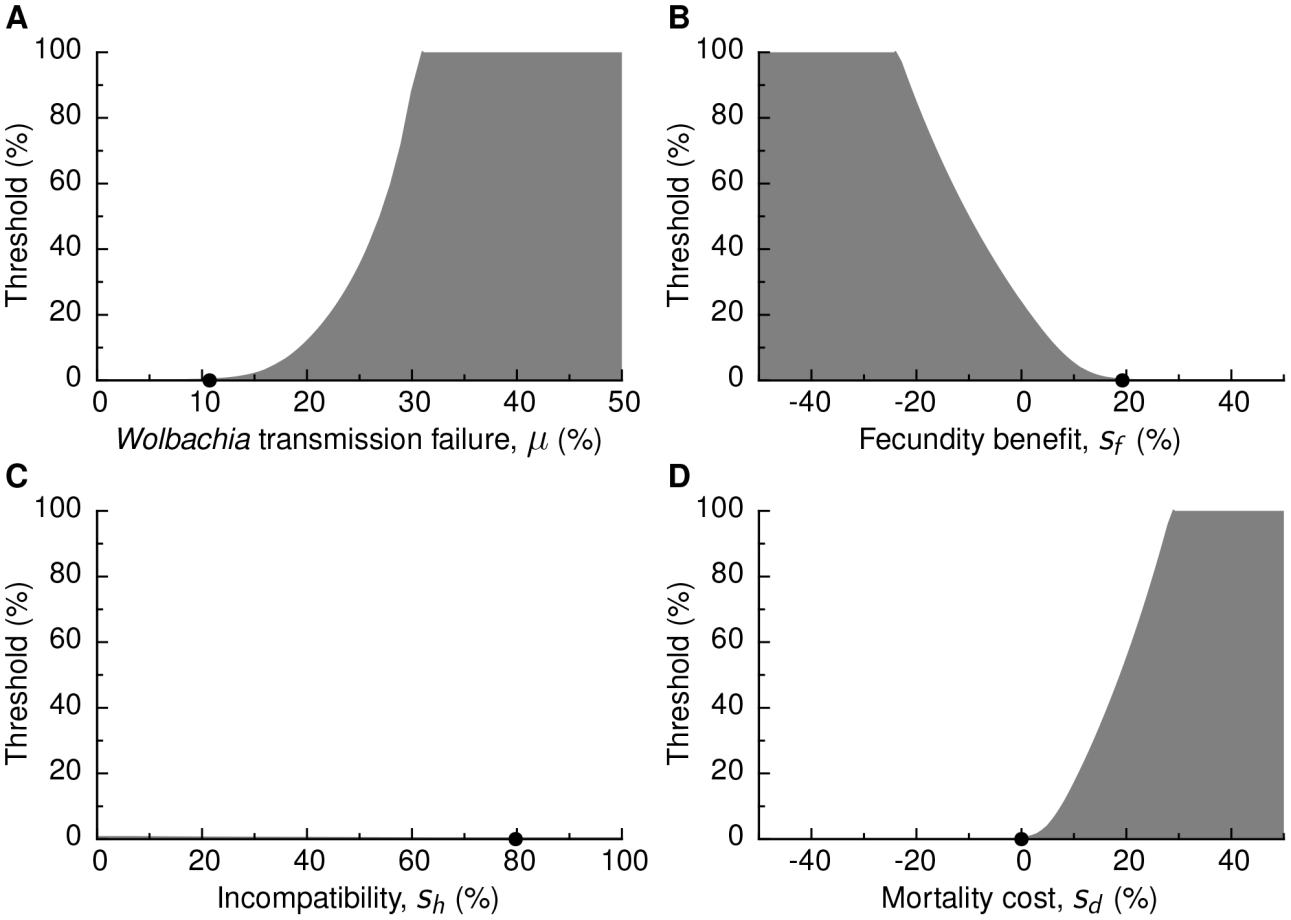
Sensitivity of paratransgene release threshold to *Wolbachia*-CI parameters. The population replacement threshold is shown for ranges of values of (A) the proportion of eggs of *Wolbachia*-colonized females that are non-colonized (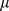), (B) increase in egg count due to colonization by *Wolbachia* (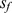), (C) the proportion of non-viable fertilizations of *Wolbachia*-colonized eggs by non-colonized sperm due to CI (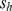), and (D) the increase in tsetse mortality associated with *Wolbachia* colonization (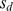). In the white region, the paratransgenic tsetse release is driven to fixation, while in the shaded region, the paratransgenic tsetse release is driven to eradication. Black points denote the baseline empirical parameter values. Empirical parameter values (Table 2) were used for parameters not varied. Note that in (C), the paratransgene is driven to fixation for all 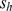 values for any size release.

For our empirical parameters, the fecundity benefit is sufficient for the fitness of *Wolbachia* to be positive even at low frequencies, such that there is no threshold: a paratransgenic release of any size will eventually lead to the fixation of the paratransgene. As a sensitivity analysis, we varied the CI parameters and examined the resulting threshold release size required to drive the paratransgene to fixation (Figure 3). Around the empirical parameter values, the threshold size is sensitive to transmission failure (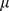), fecundity benefit (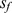) and mortality cost (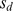), and highly insensitive to incompatibility (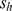). However, when transmission failure is high (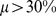), fecundity cost is high (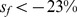), or mortality cost is high (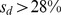), paratransgenic fixation is not feasible and, consequently, trypanosomiasis is not eliminated.

### Imperfect paratransgenic immunity

**Figure 4:**
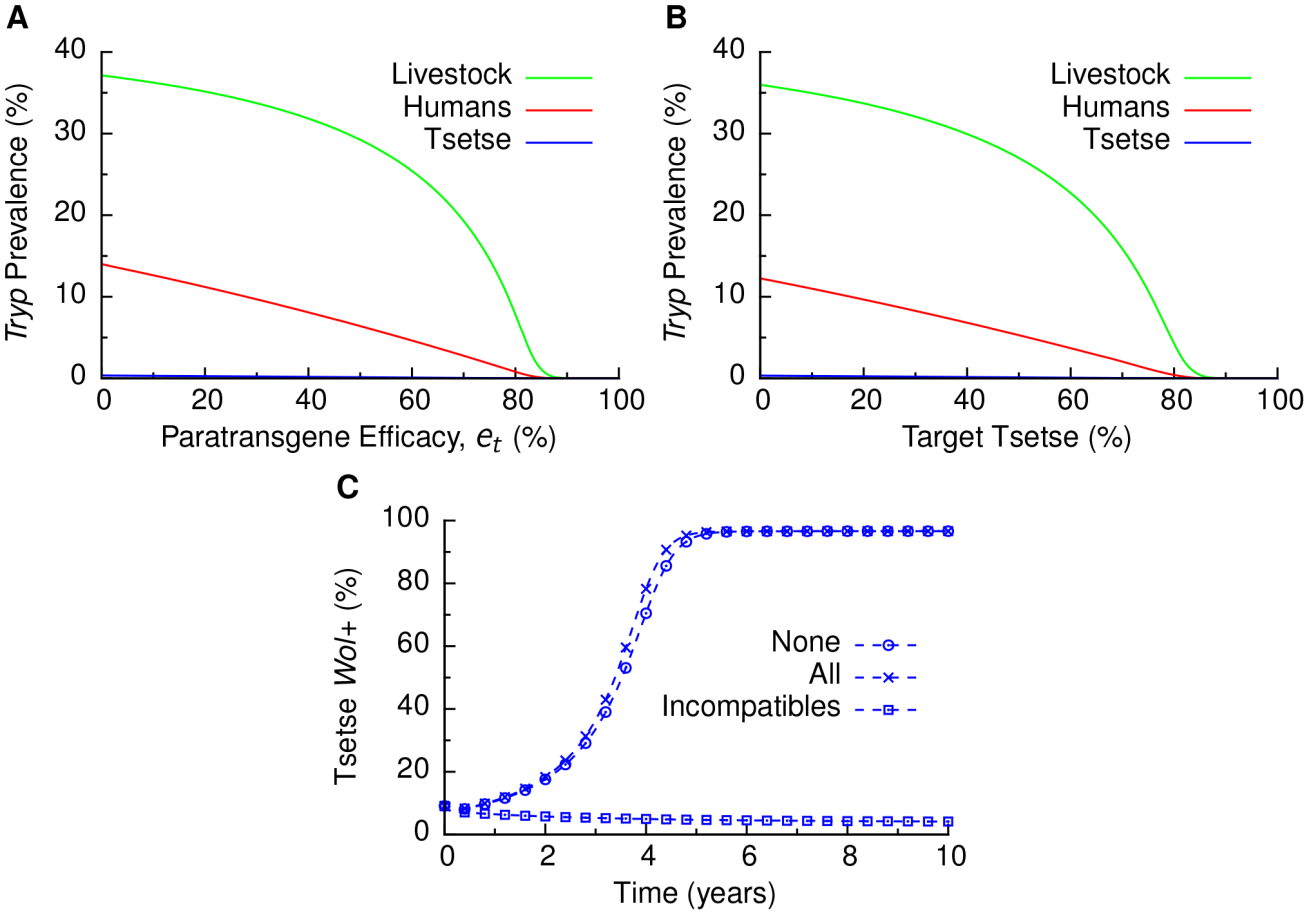
Impact of imperfect paratransgenic immunity, tsetse population composition, and tsetse remating on paratransgene effectiveness. (A) Effect of an imperfect paratransgene that provides perfect immunity to trypanosomes for proportion 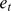 of tsetse carrying the paratransgene and no immunity to the remaining 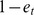 of tsetse carrying the transgene. (B) Effect of multiple tsetse species inhabiting the target region. Only the target tsetse species is affected by the paratransgene. (C) Effect of remating by no female tsetse (“None”, the baseline, same as Figure 2A), by all female tsetse (“All”), and by only female tsetse with incompatible sperm (“Incompatibles”). When remating was allowed, 38% of the eligible females mated a second time. Empirical values (Table 2) were used for the other parameters. Paratransgene releases were 10% of the wild tsetse population.

It is plausible that the paratransgene may only confer imperfect immunity against trypanosomes. To determine the impact of imperfect paratransgenic immunity against trypanosomiasis on disease prevalence, we considered the possibility that a proportion 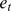 of *Wolbachia*-colonized tsetse are immune to trypanosome infection, and a proportion 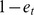 are susceptible but are still able to transmit *Wolbachia* colonization. We found that imperfect immunity does not affect population replacement by paratransgenic tsetse. However, imperfect immunity impacts trypanosomiasis prevalence among tsetse, humans, and the animal reservoirs, because trypanosome transmission is not as effectively suppressed by the paratransgene (Figure 4A). Our results suggest that imperfect immunity has little effect on the dynamics of trypanosomiasis elimination provided that the efficacy of paratransgenic immunity is above 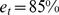. Conversely, efficacy below 85% reduces the efficacy of paratransgenic control (Figure 4A).

### Cohabitation of multiple tsetse species

Multiple species of tsetse can inhabit the same region. For example, a maximum monthly proportion of any single species of tsetse (*G. pallicera*) over several years was found to be 79% at a sampling site in Côte d'Ivoire [27]. Because intra-species mating among tsetse species results in either no offspring or sterile offspring [27], paratransgenic tsetse releases can be assumed to be species-specific. We found that when the targeted tsetse species comprises 85% or more of the tsetse population, trypanosomiasis is eliminated (Figure 4C). However, when the targeted species is a smaller fraction of the tsetse population, trypanosomiasis is reduced to a lower endemic prevalence without being eliminated. For example, if the target species is 79%, trypanosomiasis long-term prevalence is reduced more than 20 fold in humans and 6 fold in livestock.

### Tsetse remating

Evidence for remating of female tsetse is mixed. For example, one study supports the hypothesis that female tsetse typically mate only once soon after their emergence as adults and store the sperm from this mating to fertilize eggs throughout their lives [24], [28]. Conversely, the proportion of female tsetse mating more than once has been estimated to be as high as 38% [25], [29], [30]. In addition, there may be differences in remating rates between species [31]. Thus we compared our baseline scenario with a scenario where all female tsetse can remate. We also considered the possibility that failure to produce offspring due to incompatible first matings may affect the likelihood of remating among female tsetse.

We found that differences in remating rates [29] can impact the effectiveness of a paratransgenic control strategy (Figure 4C). When remating occurs equally for all females, remating does not substantially affect CI dynamics. However, if only females that have incompatible first matings can remate, *Wolbachia*-colonized tsetse invade the population more slowly than when no remating takes place and may even be unsuccessful in invading. When only incompatible females remate, if fewer than 27% remate, *Wolbachia* is driven to fixation, but more slowly than with no remating. If 27% or more of the incompatible females remate, *Wolbachia* is driven out of the population.

## Discussion

To evaluate the potential for using CI as a mechanism to drive trypanosome-refractory paratransgenes into tsetse populations, we integrated the population genetics of CI for *Wolbachia*-colonized tsetse into a dynamic model of trypanosomiasis in tsetse, humans, and an animal reservoir. Based on empirical data for CI in tsetse, we found that a one-time release of paratransgenic tsetse could eliminate trypanosomiasis, provided that extra mortality due to *Wolbachia* colonization is low, that the paratransgene is effective at protecting against trypanosome transmission, and that the target tsetse species comprises a large majority of the tsetse population in the release location.

Due to the relatively slow transient dynamics of paratransgenic population replacement, it is crucial to understand not only if trypanosomiasis can be eliminated, but also how quickly elimination occurs. Specifically, the size of the paratransgenic release determines whether the time scale of elimination is a year or a decade. For example, a release of 20% of the wild-type population would eliminate trypanosomiasis in about 4 years, while a release of 10% would take 6 years eliminate trypanosomiasis.

The best available parameter estimates are for *Wolbachia* in the tsetse *G. m. morsitans* and the trypanosome *T. b. gambiense*. The empirical *Wolbachia* CI parameters were derived from laboratory experiments on the tsetse *G. m. morsitans* [17]. Parametrizing our model with these empirical values, only a large mortality cost of *Wolbachia* colonization, insufficient immunity of the paratransgenic tsetse to trypanosomiasis transmission, or the cohabitation of multiple tsetse species could prevent the elimination of trypanosomiasis. The empirical parameter estimates include a fecundity benefit of 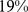 for *Wolbachia* colonization. Similar frequency-independent fitness benefits of *Wolbachia* colonization have been observed in *Drosophila melanogaster* [12] and the mosquito *Aedes albopictus* [13]. However, there are no empirical estimates of the effect of *Wolbachia* on tsetse mortality or other tsetse life-history traits. Therefore it is conceivable that *Wolbachia* colonization could have an overall negative impact on tsetse fitness when all components of fitness are incorporated. For example, *D. melanogaster* from wild populations showed weaker CI than in laboratory populations [12]. If this were true for tsetse, reduced transmission of *Wolbachia* to offspring could generate a threshold below which a release would fail, even in the presence of a frequency-independent fitness benefit of *Wolbachia* colonization. Moreover, *Wolbachia*-induced CI remains to be investigated in the many other species of tsetse that transmit trypanosomiasis to humans and livestock [24].

Our analysis shows that a multi-species tsetse population can be effectively manipulated to control trypanosomiasis provided that the target species is in the majority. A single-species paratransgenic release has the potential to prevent trypanosomiasis transmission among this species. However, if the non-targeted tsetse species in the control location are abundant, they alone may be sufficient to support trypanosomiasis endemicity, albeit at a lower prevalence. Different feeding preferences of different tsetse species cohabiting the same area may create separate epidemiological systems that maintain trypanosomes: in this setting, eliminating trypanosomes from one tsetse species would not eliminate trypanosomes from the whole area, leaving humans at risk. Moreover, differences in trapping efficiency of tsetse species may result in unreliable estimates of the relative size of the species' populations. Controlling multiple tsetse species by paratransgenesis could be difficult as *Wolbachia* drivers and transgenic *Sodalis* must be developed for each species. In contrast, differences in competence for trypanosome transmission to humans and livestock may mitigate the importance of many tsetse species. More research is needed to understand the control of trypanosomes in areas with multiple tsetse species.

It is possible that the trypanosome-refractory transgene in the symbiont *Sodalis* could become unlinked from the *Wolbachia* used to drive the paratransgene into the tsetse population [9], [32]. In addition, some wild populations of tsetse already harbor *Wolbachia* [15], which may interfere with the colonization of a new strain of *Wolbachia*. However, if the new strain exhibits bidirectional CI with the established resident strain, whereby eggs colonized by the each strain are incompatible with sperm from males colonized by the other strain, then it could drive paratransgenic tsetse into the population, provided a sufficiently large release [33]. In addition, a potential anti-trypanosome effect of *Wolbachia* colonization, which we have not included in our model, would increase the effectiveness of the paratransgene and perhaps also mitigate for the paratransgene becoming unlinked *Wolbachia*. This possibility is supported by the observation that *Wolbachia* colonization in *Aedes aegypti* mosquitoes activates an immune response that protects the mosquitoes against infection with dengue and other vector-borne pathogens [34]–[37]. A similar immune response to *Wolbachia* has also been seen in *Drosophila* [38].

Trypanosome infection has been observed to have a fitness cost to tsetse [39], but the very low prevalence of trypanosomes in tsetse populations means that trypanosomes would not play a substantial role in selection for resistant paratransgenes in the few generations until establishment of a successful introduction. Indeed, we made the conservative assumption that there was no tsetse fitness cost from trypanosome infection, although the results would likely be very similar using e.g. the estimate of a 30% fecundity cost from trypanosome infection [39].

Trypanosomiasis control policies have focused on reducing tsetse density with insecticide and, in some cases, sterile-insect technique. Given that seasonal fluctuations in the abundance of different tsetse species could compromise the speed or viability of tsetse population replacement [27], combinations of controls measures, such as treatment of animal reservoir hosts as well as control of wild game or habitat disruption [24], could synergistically enhance the efficacy of a paratransgene release and speed the eradication of trypanosomes from a target area. Unlike conventional tsetse control, *Wolbachia*-induced CI could prevent trypanosomiasis-tolerant tsetse from re-invading treated areas and could allow paratransgenic tsetse to invade and replace neighboring populations. Indeed, it might be possible to release paratransgenic tsetse into a target area and then, once the paratransgene is driven to fixation, capture paratransgenic tsetse from this area to release in the next target area, saving on-going costs of colony breeding.

In our modeling, we have assumed that once trypanosome-refractory tsetse are established, they do not lose effectiveness over time. Effectiveness would wane if the transgenic *Sodalis* becomes unlinked from the *Wolbachia* driver, e.g. if maternal transmission of *Sodalis* is not perfect. Over time, transgenic *Sodalis* could shed the trypanosome-resistant genes or trypanosomes could develop resistance. Should effectiveness wane, the control area may be able to again sustain trypanosome transmission. Our analysis has only examined whether paratransgenic tsetse can be effective without waning effectiveness: in future work we will closely examine the potential for waning, the time until the tsetse population reverts to transmission potential, and the resulting potential effectiveness over time of paratransgenic tsetse.

Like sterile-insect technique, where irradiated males are released to decrease females' mating success, this paratransgenic strategy would require breeding large numbers of tsetse in a colony for release. Unlike irradiated males, we believe that paratransgenesis is unlikely to cause high mortality or low mobility as both *Wolbachia* and *Sodalis* occur in wild tsetse populations, although this will have to be established empirically in laboratory and wild populations. Efficient colony breeding may require releasing more males than females, while, in our analysis, we assumed that paratransgenic releases have a balanced sex ratio. Of course, paratransgenic releases must contain some females as only they transmit *Wolbachia* to their offspring, which in turn drives the trypanosome-resistant transgene into the population. If breeding necessitates male-biased paratransgeneic releases, a balanced sex ratio would be reestablished after the first generation provided that the releases were not too heavily biased towards males, and our model predictions would not change. In addition, ethical concerns over releasing tsetse should be less for paratransgenesis than for sterile-insect technique as the paratransgenic tsetse are resistant to trypanosome transmission.

We developed a mathematical model that integrates *Wolbachia* population genetics and trypanosomiasis epidemiology, parametrized by recent empirical studies of *Wolbachia* in tsetse. We used this modeling framework to evaluate a novel paratransgenic control strategy to eliminate trypanosomiasis and provide predictions of feasibility and speed of trypanosomiasis elimination. We find that paratransgenesis has the potential to be a feasible strategy to control trypanosomiasis rapidly.

## Supporting Information

Text S1 **Detailed modeling methods.**
(PDF)
