# Supplementary material for: Evaluating Paratransgenesis as a Potential Control Strategy for African Trypanosomiasis
Source: PLoS Negl Trop Dis. 2013 Aug 15;7(8):e2374. doi: 10.1371/journal.pntd.0002374 (PMC3744416; doi:10.1371/journal.pntd.0002374)
Supplement: Text S1 — Detailed modeling methods. (PDF) [file pntd.0002374.s001.pdf]

# *Detailed modeling methods for* Evaluating paratransgenesis as a potential control strategy for African trypanosomiasis

Jan Medlock[1,2*], Katherine E. Atkins[2], David N. Thomas[2], Serap Aksoy[2], Alison P. Galvani[2]
**1 Department of Biological Sciences, Oregon State University, 105 Magruder Hall, Corvallis, Oregon 97331, USA**
**2 Yale School of Public Health, 135 College Street, New Haven, Connecticut 06510, USA**
**∗ E-mail: jan.medlock@oregonstate.edu**

# Model construction

To determine the potential of a trypanosome-refractory paratransgenic intervention to combat trypanosomiasis, we developed a continuous-time CI model based on existing discrete-time models, and then integrated this into a three-species SEIR model of CI and trypanosomiasis among tsetse, humans, and animal reservoirs. This model was extended to incorporate imperfect paratransgene-mediated resistance, multiple tsetse species, and remating by female tsetse.

## An overlapping-generations, continuous-time CI model

Based on our previous modeling work [1], we developed a continuous-time model for *Wolbachia* colonization in tsetse that served as the basis for our age-structured model. This model was not used to generate the results in this work: we present it here solely to aid in the understanding of the age-structured model that was used. Let $V_p(t)$ be the number of *Wolbachia*-colonized tsetse at time $t$, $V_n(t)$ be the number of non-colonized tsetse at time $t$, and $V(t) = V_p(t) + V_n(t)$, then the model is given by

$$\frac{\mathrm{d}V_p}{\mathrm{d}t} = r(1+s_f)(1-\mu)\left(\frac{V_p}{V}+\frac{V_n}{V}\right)V_p - d(1+s_d)V_p, \tag{S1a}$$

$$\begin{aligned}\frac{\mathrm{d}V_n}{\mathrm{d}t} = {} & r(1+s_f)\mu\left[(1-s_h)\frac{V_p}{V}+\frac{V_n}{V}\right]V_p \\ & + r\left[(1-s_h)\frac{V_p}{V}+\frac{V_n}{V}\right]V_n - dV_n.\end{aligned} \tag{S1b}$$

The first term on the right-hand side of (S1a) is for birth of *Wolbachia*-colonized offspring: eggs are produced by colonized females at rate $r(1+s_f)V_p$, the proportion $1-\mu$ of those eggs are colonized, and then they are fertilized with 100% efficacy by either colonized or non-colonized males, represented by the term $V_p/V + V_n/V$. In (S1b), the first and second terms on the right-hand side are birth of non-colonized offspring from colonized females and from non-colonized females, respectively. Non-colonized eggs are produced at rates $r(1+s_f)\mu V_p$ by colonized females and $rV_n$ by non-colonized females; non-colonized eggs are then fertilized with success $1-s_h$ by colonized males and with 100% success by non-colonized males. Finally, the last two terms in both right-hand sides are for death.

Simplifying these two equations gives

$$\frac{\mathrm{d}V_p}{\mathrm{d}t} = [r(1+s_f)(1-\mu) - d(1+s_d)]V_p, \tag{S2a}$$

$$\frac{\mathrm{d}V_n}{\mathrm{d}t} = r\left(1-s_h\frac{V_p}{V}\right)[\mu(1+s_f)V_p + V_n] - dV_n. \tag{S2b}$$

They can be simplified further by considering $p(t) = V_p(t)/V(t)$, the proportion of tsetse that are *Wolbachia* colonized, giving

$$\frac{\mathrm{d}p}{\mathrm{d}t} = \frac{1}{V}\frac{\mathrm{d}V_p}{\mathrm{d}t} - \frac{V_p}{V^2}\frac{\mathrm{d}V}{\mathrm{d}t} = \frac{1}{V}\left(\frac{\mathrm{d}V_p}{\mathrm{d}t} - p\frac{\mathrm{d}V}{\mathrm{d}t}\right), \tag{S3}$$

where

$$\frac{\mathrm{d}V}{\mathrm{d}t} = \frac{\mathrm{d}}{\mathrm{d}t}(V_p + V_n) = \frac{\mathrm{d}V_p}{\mathrm{d}t} + \frac{\mathrm{d}V_n}{\mathrm{d}t}. \tag{S4}$$

Thus, using (S2) and $V_n/V = 1 - p$ results in the single differential equation for the proportion of tsetse that are *Wolbachia* colonized

$$\begin{aligned}\frac{\mathrm{d}p}{\mathrm{d}t} = -p\Big(&\big\{r[1-(1+s_f)(1-\mu)] + s_d d\big\} \\ &- [r(s_h - s_f) + s_d d]p + r s_h[1-\mu(1+s_f)]p^2\Big).\end{aligned} \tag{S5}$$

In general, this model has equilibria with no *Wolbachia* colonization in the population, $p_0 = 0$, and with a fixation level of *Wolbachia* in the population, $p_F$ with $p_0 \leq p_F < 1$. It may also have an unstable equilibrium $p_T$ with $p_0 \leq p_T \leq p_F$. The model has 3 different kinds of qualitative behavior, depending on the parameter values:

1. *Wolbachia* cannot persist in the population. $p_0 = p_F = 0$ is the only equilibrium and it is stable.
2. The presence of any amount of *Wolbachia* leads to fixation. $p_0 = p_T = 0$ is unstable and $p_F$ is stable with $p_0 < p_F < 1$.
3. There is a threshold $p_T$ with $p_0 < p_T < p_F$. $p_T$ is unstable, and $p_0$ and $p_F$ are stable. Initial levels of colonization below $p_T$ result in *Wolbachia* being removed from the population ($p(t) \to p_0$), while initial levels of colonization above $p_T$ result in fixation of *Wolbachia* ($p(t) \to p_F$).

When considering the release of transgenic tsetse driven by *Wolbachia* for trypanosomiasis control, $p_T$ gives the release threshold, the size that the release must be in order to result in fixation of the transgene. See Supporting Information Text S1 of [1] for more information[1].

To derive the parameters of this continuous-time model from the discrete-time parameters (Table 2), we compared the two linear models

$$\frac{\mathrm{d}V}{\mathrm{d}t} = rV - d(1+s_d)V \qquad \Longrightarrow \qquad V(t) = \mathrm{e}^{[r-d(1+s_d)]t}V(0), \tag{S6}$$

$$v_{t+1} = m v_t + \rho(1-s_\rho)v_t \qquad \Longrightarrow \qquad v_t = [m + \rho(1-s_\rho)]^t v_0. \tag{S7}$$

Setting $V(t) = v_t$ results in

$$d = -\log(\rho), \qquad r = \log\left(1 + \frac{m}{\rho}\right), \qquad s_d = \frac{\log(1-s_\rho)}{\log(\rho)}. \tag{S8}$$

## A continuous-time, discrete-age three-species SEIR model of trypanosomiasis and *Wolbachia* colonization

For *Trypanosoma brucei gambiense*, which causes 95% of reported human cases of trypanosomiasis [2], tsetse are only infected during their first bloodmeal, within 24 hours after emergence [3]. Rogers [4] found that *T. brucei* infection in humans could not be sustained without animal reservoirs. Consequently, we included both humans and animal reservoirs in our model, and parameterized our model to that "'typical' of a village situation in West Africa" formulated by Rogers [4].

Considering these features of trypanosomiasis in humans, we developed a three-species, discrete-age, continuous-time model of CI and trypanosome infection that captures *T. brucei* transmission dynamics

[1] In our previous work [1], we separated the rate of offspring production (there called $r$) and the survival of that offspring ($f$ there), each with their own relative fitness term for *Wolbachia* colonization. With no loss of generality, here we combined both rate of offspring production and offspring survival into the single parameter $r$ and the corresponding relative fitness terms are combined into $s_f$.

and explicitly tracks *Wolbachia*-prevalence at time of mating. We divided the tsetse lifecycle into 13 age classes, each 10 days long, starting with deposit as a pupa [5]. Emergence from pupa to adult and first blood meal were assumed to occur as tsetse enters the sixth age class ($i_A = 6$, after 50–59 days after deposit as pupa). Egg laying was assumed to begin in the seventh age class (60–69 days after deposit as pupa).

We assumed that the number of male tsetse are proportional to the number of females in each *Wolbachia* status and age class, i.e. the same constant of proportionality in each status and age class, so that we need not track females and males separately. Let subscript $n$ denote non-*Wolbachia*-colonized tsetse and subscript $p$ denote *Wolbachia*-colonized tsetse. Let the vector $\mathbf{V}_{pn}$ be the number of *Wolbachia*-colonized female tsetse in each age class that have not mated with *Wolbachia*-colonized male tsetse and the vector $\mathbf{V}_{pp}$ be the number of *Wolbachia*-colonized female tsetse in each age class that have mated with *Wolbachia*-colonized male tsetse. Likewise, let the vector $\mathbf{V}_{nn}$ denote non-colonized female tsetse in each age class that have not mated with *Wolbachia*-colonized male tsetse, and the vector $\mathbf{V}_{np}$ denote non-colonized female tsetse in each age class that have mated with *Wolbachia*-colonized male tsetse. Note that $\mathbf{V}_{pn}$ and $\mathbf{V}_{nn}$ count both females that have not mated and females that have mated with non-colonized males. Subscripts $S$, $E$, $I$, and $R$ denote susceptible, exposed, infected, and recovered trypanosomiasis status, so then non-*Wolbachia*-colonized female tsetse populations are given by

$$\mathbf{V}_{nn} = \mathbf{V}_{nnS} + \mathbf{V}_{nnE} + \mathbf{V}_{nnI} + \mathbf{V}_{nnR}, \tag{S9a}$$

$$\mathbf{V}_{np} = \mathbf{V}_{npS} + \mathbf{V}_{npE} + \mathbf{V}_{npI} + \mathbf{V}_{npR}. \tag{S9b}$$

The model equations describing the change in tsetse numbers are

$$\begin{aligned}\frac{\mathrm{d}\mathbf{V}_{pn}}{\mathrm{d}t} &= \mathbf{B}_p\left(\mathbf{V}_{pn} + \mathbf{V}_{pp}\right) + \mathbf{A}\mathbf{V}_{pn} - \mathbf{D}_p\mathbf{V}_{pn} \\ &\quad - \phi\mathbf{M}\mathbf{V}_{pn} + (1-\phi)\theta_c\mathbf{R}\mathbf{V}_{pp} - \phi\theta_c\mathbf{R}\mathbf{V}_{pn},\end{aligned} \tag{S10a}$$

$$\begin{aligned}\frac{\mathrm{d}\mathbf{V}_{pp}}{\mathrm{d}t} &= \mathbf{A}\mathbf{V}_{pp} - \mathbf{D}_p\mathbf{V}_{pp} + \phi\mathbf{M}\mathbf{V}_{pn} \\ &\quad - (1-\phi)\theta_c\mathbf{R}\mathbf{V}_{pp} + \phi\theta_c\mathbf{R}\mathbf{V}_{pn},\end{aligned} \tag{S10b}$$

$$\begin{aligned}\frac{\mathrm{d}\mathbf{V}_{nnS}}{\mathrm{d}t} &= \mathbf{B}_{nn}\left(\mathbf{V}_{nn} - \epsilon_V\mathbf{V}_{nnI}\right) + \mathbf{B}_{np}\left(\mathbf{V}_{np} - \epsilon_V\mathbf{V}_{npI}\right) \\ &\quad + \mathbf{B}_{pn}\mathbf{V}_{pn} + \mathbf{B}_{pp}\mathbf{V}_{pp} \\ &\quad + \mathbf{A}\mathbf{V}_{nnS} - \mathbf{D}_n\mathbf{V}_{nnS} - (a_H + a_L + v)\mathbf{V}_{nnS} \\ &\quad - \phi\mathbf{M}\mathbf{V}_{nnS} + (1-\phi)\theta_i\mathbf{R}\mathbf{V}_{npS} - \phi\theta_c\mathbf{R}\mathbf{V}_{nnS},\end{aligned} \tag{S10c}$$

$$\begin{aligned}\frac{\mathrm{d}\mathbf{V}_{npS}}{\mathrm{d}t} &= \mathbf{A}\mathbf{V}_{npS} - \mathbf{D}_n\mathbf{V}_{npS} + \phi\mathbf{M}\mathbf{V}_{nnS} - (a_H + a_L + v)\mathbf{V}_{npS} \\ &\quad - (1-\phi)\theta_i\mathbf{R}\mathbf{V}_{npS} + \phi\theta_c\mathbf{R}\mathbf{V}_{nnS},\end{aligned} \tag{S10d}$$

$$\begin{aligned}\frac{\mathrm{d}\mathbf{V}_{nnE}}{\mathrm{d}t} &= \mathbf{A}\mathbf{V}_{nnE} - \mathbf{D}_n\mathbf{V}_{nnE} - \tau_V\mathbf{V}_{nnE} - \phi\mathbf{M}\mathbf{V}_{nnE} \\ &\quad + (a_H\lambda_{VH} + a_L\lambda_{VL})\mathbf{V}_{nnS} \\ &\quad + (1-\phi)\theta_i\mathbf{R}\mathbf{V}_{npE} - \phi\theta_c\mathbf{R}\mathbf{V}_{nnE},\end{aligned} \tag{S10e}$$

$$\begin{aligned}\frac{\mathrm{d}\mathbf{V}_{npE}}{\mathrm{d}t} &= \mathbf{A}\mathbf{V}_{npE} - \mathbf{D}_n\mathbf{V}_{npE} + \phi\mathbf{M}\mathbf{V}_{nnE} + (a_H\lambda_{VH} + a_L\lambda_{VL})\mathbf{V}_{npS} \\ &\quad - \tau_V\mathbf{V}_{npE} - (1-\phi)\theta_i\mathbf{R}\mathbf{V}_{npE} + \phi\theta_c\mathbf{R}\mathbf{V}_{nnE},\end{aligned} \tag{S10f}$$

$$\begin{aligned}\frac{\mathrm{d}\mathbf{V}_{nnI}}{\mathrm{d}t} &= \mathbf{A}\mathbf{V}_{nnI} - \mathbf{D}_n\mathbf{V}_{nnI} + \tau_V\mathbf{V}_{nnE} \\ &\quad - \phi\mathbf{M}\mathbf{V}_{nnI} + (1-\phi)\theta_i\mathbf{R}\mathbf{V}_{npI} - \phi\theta_c\mathbf{R}\mathbf{V}_{nnI},\end{aligned} \tag{S10g}$$

$$\frac{\mathrm{d}\mathbf{V}_{npI}}{\mathrm{d}t} = \mathbf{A}\mathbf{V}_{npI} - \mathbf{D}_n\mathbf{V}_{npI} + \phi\mathbf{M}\mathbf{V}_{nnI} + \tau_V\mathbf{V}_{npE} - (1-\phi)\theta_i\mathbf{R}\mathbf{V}_{npI} + \phi\theta_c\mathbf{R}\mathbf{V}_{nnI}, \quad \text{(S10h)}$$

$$\frac{\mathrm{d}\mathbf{V}_{nnR}}{\mathrm{d}t} = \mathbf{A}\mathbf{V}_{nnR} - \mathbf{D}_n\mathbf{V}_{nnR} + \left[a_H(1-\lambda_{VH}) + a_L(1-\lambda_{VL}) + v\right]\mathbf{V}_{nnS} - \phi\mathbf{M}\mathbf{V}_{nnR} + (1-\phi)\theta_i\mathbf{R}\mathbf{V}_{npR} - \phi\theta_c\mathbf{R}\mathbf{V}_{nnR}, \quad \text{(S10i)}$$

$$\frac{\mathrm{d}\mathbf{V}_{npR}}{\mathrm{d}t} = \mathbf{A}\mathbf{V}_{npR} - \mathbf{D}_n\mathbf{V}_{npR} + \left[a_H(1-\lambda_{VH}) + a_L(1-\lambda_{VL}) + v\right]\mathbf{V}_{npS} + \phi\mathbf{M}\mathbf{V}_{nnR} - (1-\phi)\theta_i\mathbf{R}\mathbf{V}_{npR} + \phi\theta_c\mathbf{R}\mathbf{V}_{nnR}. \quad \text{(S10j)}$$

The probabilities of a susceptible tsetse becoming infected from a blood meal are

$$\lambda_{VH} = \beta_{VH}\frac{H_I}{H}, \quad \text{(S11a)}$$

$$\lambda_{VL} = \beta_{VL}\frac{L_I}{L}. \quad \text{(S11b)}$$

Births are given by

$$\mathbf{B}_\circ = \begin{bmatrix} \leftarrow & \mathbf{b}_\circ & \rightarrow \\ 0 & \dots & 0 \\ \vdots & & \vdots \\ 0 & \dots & 0 \end{bmatrix}, \quad \text{for } \circ = nn, np, pn, pp, \text{and } p, \quad \text{(S12)}$$

where

$$\mathbf{b}_{nn} = Z(V)\mathbf{r}, \quad \text{(S13a)}$$

$$\mathbf{b}_{np} = Z(V)(1-\mathbf{s}_h)\otimes\mathbf{r}, \quad \text{(S13b)}$$

$$\mathbf{b}_{pn} = Z(V)\mu(1+s_f)\mathbf{r}, \quad \text{(S13c)}$$

$$\mathbf{b}_{pp} = Z(V)\mu(1+s_f)(1-\mathbf{s}_h)\otimes\mathbf{r}, \quad \text{(S13d)}$$

$$\mathbf{b}_{p} = Z(V)(1-\mu)(1+s_f)\mathbf{r}; \quad \text{(S13e)}$$

$\otimes$ signifies element-by-element matrix multiplication (the Hadamard product); $\epsilon_V$ is the fecundity cost to female tsetse of trypanosome infection, which we assume to be 0 (although see Hu et al. [6], where $\epsilon_V$ was estimated to be 30%); the vector of fecundity rates is

$$\mathbf{r} = [r_i], \qquad r_i = \begin{cases} 0 & \text{if } i \le i_A, \\ \log(1+m) & \text{if } i > i_A; \end{cases} \quad \text{(S14)}$$

such that females start having offspring in the next age class after mating, i.e. 60–69 days after deposit as pupa; and $Z(V)$ is a decreasing function of tsetse population size that prevents unrealistic exponential population growth. Tsetse are known to have relatively stable population sizes, yet the mechanisms of population regulation are unclear [3]. Thus we choose the simple linearly decreasing function

$$Z(V) = 1 - \frac{V}{K_V}, \quad \text{(S15)}$$

where

$$\mathbf{V} = \mathbf{V}_{pn} + \mathbf{V}_{pp} + \mathbf{V}_{nn} + \mathbf{V}_{np},$$
$$V = \sum_a V_a \tag{S16}$$

is the total tsetse population size. The scaling constant $K_V$ gives $Z(K_V) = 0$ so that all the birth rates are 0: thus $K_V$ is an upper bound on the equilibrium tsetse population size. We chose $K_V = 16538$ so that the equilibrium tsetse population size in the absence of *Wolbachia* is 5000 as in Rogers's typical West African village. The non-zero elements of $\mathbf{B}_\circ$ are all in the first row because all offspring are born into the first age class.

Aging is given by

$$\mathbf{A} = \begin{bmatrix} \nwarrow & 0 & \dots & 0 \\ \nwarrow & -\alpha & \ddots & \vdots \\ 0 & \alpha & \searrow & 0 \\ 0 & 0 & \searrow & 0 \end{bmatrix}, \tag{S17}$$

where $\alpha = 1/10$ day$^{-1}$ is the transition rate from one age class to the next older age class after an average stay of 10 days, with the exception of the last age class, which accumulates all tsetse aged 120 or more days after deposit as pupa.

Deaths are given by the rates

$$\mathbf{D}_n = \mathbf{D}, \tag{S18a}$$
$$\mathbf{D}_p = (1 + s_d)\mathbf{D}, \tag{S18b}$$

with the proportional increase in mortality $s_d$ to *Wolbachia*-colonized tsetse and

$$\mathbf{D} = [d_{ij}],$$
$$d_{ij} = \begin{cases} -\log(\rho_P) & \text{if } i = j < i_A, \\ -\log(\rho) & \text{if } i = j \geq i_A, \\ 0 & \text{if } i \neq j, \end{cases} \tag{S19}$$

using separate pupal and adult survivals.

Female tsetse generally mate once, after their first bloodmeal and within the first thirteen days after adult emergence. Females carry sperm from this mating for life, although remating has been observed among as many as 38% of females [3,4,7]. Since we assumed that the number of males is proportional to the number of females in each age class, the proportion of matings by *Wolbachia*-colonized males is

$$\phi = \frac{\mathbf{q}_m^T (\mathbf{V}_{pn} + \mathbf{V}_{pp})}{\mathbf{q}_m^T \mathbf{V}}, \tag{S20}$$

where $\mathbf{q}_m^T$ is the relative fecundity of each male age class. We data we used to parametrize male fecundity ( [8]; Table S1) excludes competition between males. Although this is admittedly a poor proxy for mating intensity, it provides some empirical basis for waning mating success with male age. These data were extrapolated to the model's 10-day age classes (Table S2). The mating matrix is given by

$$\mathbf{M} = [m_{ij}], \tag{S21a}$$

with

$$m_{ij} = \begin{cases} \alpha & \text{if } i = i_A + 1,\ j = i_A, \\ 0 & \text{otherwise,} \end{cases} \tag{S21b}$$

**Table S1.** Data on male mating success versus age from [8]. Males aged according to the column on the left were allowed to mate with newly emerged females, which resulted in proportions of females producing offspring given by the column on the right. See also Table S2.

| Days since emergence as adult | Insemination success |
|---|---|
| 6–14 | 96.5% |
| 13–21 | 98.2% |
| 20–28 | 99.2% |
| 27–35 | 93.3% |
| 34–42 | 62.7% |
| 41–49 | 21.5% |
| 48–56 | 2.0% |
| 55–98 | 0.0% |

where $i_A = 6$ is the initial female mating age (50–59 days after deposit as pupa), which moves females from unmated in age class 6 to mated in age class 7, at the same rate as aging ($\alpha$) so that all females are mated over the duration of this age class. The fraction $\phi$ mate with *Wolbachia*-colonized, moving from $\mathbf{V}_{pn}$ to $\mathbf{V}_{pp}$ or $\mathbf{V}_{nn\circ}$ to $\mathbf{V}_{np\circ}$ (for $\circ = S, E, I, R$), while the remaining fraction $1 - \phi$ mate with non-*Wolbachia*-colonized males, remaining in $\mathbf{V}_{pn}$ or $\mathbf{V}_{nn\circ}$. This gives rise to terms like $\mp\phi\mathbf{M}\mathbf{V}_{pn}$ in (S10a & S10b).

Paratransgene releases were simulated by first running the model with only non-*Wolbachia*-colonized tsetse present ($\mathbf{V}_{pn} = \mathbf{V}_{pp} = \mathbf{V}_{npS} = \mathbf{V}_{npE} = \mathbf{V}_{npI} = \mathbf{V}_{npR} = 0$) to equilibrium to represent the wild-type population. The initial release of paratransgenic tsetse was assumed to be comprised entirely of newly emerged adults ($a = 1$), with a sex ratio the same as that in the wild-type population, e.g. not comprised of entirely females or entirely males. The initial release of size $s$ relative to the wild-type population size was added to the equilibrium wild-type population, so that the initial population is of size $1 + s$ relative to the wild-type population. Thus, the initial condition was

$$\begin{aligned}
V_{pn,1}(0) &= sV^*, \\
V_{pn,a}(0) &= 0, && \text{for } a = 2, 3, \ldots, 13, \\
\mathbf{V}_{pp}(0) &= 0, \\
\mathbf{V}_{nn\circ}(0) &= \mathbf{V}^*_{nn\circ}, && \text{for } \circ = S, E, I, R, \\
\mathbf{V}_{np\circ}(0) &= 0, && \text{for } \circ = S, E, I, R.
\end{aligned} \tag{S22}$$

## Imperfect paratransgenic immunity

To examine the effect of imperfect immunity conferred by the *Wolbachia*-driven paratransgene on trypanosomiasis prevalence, we added a parameter to our model, $e_t$, that describes the proportion of paratransgenic tsetse that are fully immune to trypanosome infection: i.e. $e_t$ is the paratransgenic efficacy and the proportion of paratransgenic tsetse fully susceptible to trypanosome infection is $1 - e_t$.

Paratransgenic tsetse that are fully susceptible to trypanosomes now enter the susceptible compartment in our SEIR model of trypanosomiasis, but are still able to transmit the paratransgene to offspring, and are still affected by the *Wolbachia* CI fecundity factors (main text Figure 3). From the vectors of *Wolbachia*-colonized females, $\mathbf{V}_{pn}$ and $\mathbf{V}_{pp}$, in (S10), the proportion $1 - e_t$ can be infected with trypanosomes, and so must be divided into $S$, $E$, $I$, and $R$ trypanosomiasis compartments. To capture this,

**Table S2.** Male mating success versus age, $\mathbf{q}_m$. Data on male mating success (Table S1) were extrapolated into the model's 10-day age classes.

| Age class | Days since deposit as pupa | Days since emergence as adult | Insemination success, $\mathbf{q}_m$ |
|---|---|---|---|
| $i < 6$ | 0–49 | $< 0$ | 0% |
| $i = 6, 7, 8$ | 50–79 | 0–29 | 99% |
| $i = 9$ | 80–89 | 30–39 | 62% |
| $i = 10$ | 90–99 | 40–49 | 17% |
| $i = 11$ | 100–109 | 50–59 | 2% |
| $i > 11$ | $> 109$ | $> 59$ | 0% |

we removed equations (S10a & S10b) and added the following equations to the remainder of system (S10 & S34):

$$\frac{\mathrm{d}\mathbf{V}_{pnS}}{\mathrm{d}t} = (1-e_t)\mathbf{B}_p[\mathbf{V}_{pn}+\mathbf{V}_{pp}] + \mathbf{A}\mathbf{V}_{pnS} - \mathbf{D}_p\mathbf{V}_{pnS} - (a_H + a_L + v)\mathbf{V}_{pnS} - \phi\mathbf{M}\mathbf{V}_{pnS}, \tag{S23a}$$

$$\frac{\mathrm{d}\mathbf{V}_{ppS}}{\mathrm{d}t} = \mathbf{A}\mathbf{V}_{ppS} - \mathbf{D}_n\mathbf{V}_{ppS} + \phi\mathbf{M}\mathbf{V}_{pnS} - (a_H + a_L + v)\mathbf{V}_{ppS}, \tag{S23b}$$

$$\frac{\mathrm{d}\mathbf{V}_{pnE}}{\mathrm{d}t} = \mathbf{A}\mathbf{V}_{pnE} - \mathbf{D}_n\mathbf{V}_{pnE} - \tau_V\mathbf{V}_{pnE} - \phi\mathbf{M}\mathbf{V}_{pnE} + (a_H\lambda_{VH} + a_L\lambda_{VL})\mathbf{V}_{pnS}, \tag{S23c}$$

$$\frac{\mathrm{d}\mathbf{V}_{ppE}}{\mathrm{d}t} = \mathbf{A}\mathbf{V}_{ppE} - \mathbf{D}_n\mathbf{V}_{ppE} + \phi\mathbf{M}\mathbf{V}_{pnE} + (a_H\lambda_{VH} + a_L\lambda_{VL})\mathbf{V}_{ppS} - \tau_V\mathbf{V}_{ppE}, \tag{S23d}$$

$$\frac{\mathrm{d}\mathbf{V}_{pnI}}{\mathrm{d}t} = \mathbf{A}\mathbf{V}_{pnI} - \mathbf{D}_n\mathbf{V}_{pnI} + \tau_V\mathbf{V}_{pnE} - \phi\mathbf{M}\mathbf{V}_{pnI}, \tag{S23e}$$

$$\frac{\mathrm{d}\mathbf{V}_{ppI}}{\mathrm{d}t} = \mathbf{A}\mathbf{V}_{ppI} - \mathbf{D}_n\mathbf{V}_{ppI} + \phi\mathbf{M}\mathbf{V}_{pnI} + \tau_V\mathbf{V}_{ppE}, \tag{S23f}$$

$$\frac{\mathrm{d}\mathbf{V}_{pnR}}{\mathrm{d}t} = e_t\mathbf{B}_p[\mathbf{V}_{pn}+\mathbf{V}_{pp}] + \mathbf{A}\mathbf{V}_{pnR} - \mathbf{D}_n\mathbf{V}_{pnR} + [a_H(1-\lambda_{VH}) + a_L(1-\lambda_{VL}) + v]\,\mathbf{V}_{pnS} - \phi\mathbf{M}\mathbf{V}_{pnR}, \tag{S23g}$$

$$\frac{\mathrm{d}\mathbf{V}_{ppR}}{\mathrm{d}t} = \mathbf{A}\mathbf{V}_{ppR} - \mathbf{D}_n\mathbf{V}_{ppR} + [a_H(1-\lambda_{VH}) + a_L(1-\lambda_{VL}) + v]\,\mathbf{V}_{ppS} + \phi\mathbf{M}\mathbf{V}_{pnR}, \tag{S23h}$$

with

$$\mathbf{V}_{pn} = \mathbf{V}_{pnS} + \mathbf{V}_{pnE} + \mathbf{V}_{pnI} + \mathbf{V}_{pnR}, \tag{S23i}$$

$$\mathbf{V}_{pp} = \mathbf{V}_{ppS} + \mathbf{V}_{ppE} + \mathbf{V}_{ppI} + \mathbf{V}_{ppR}. \tag{S23j}$$

For simplicity, these equations do not include remating, which was not considered (i.e. $\theta_c = \theta_i = 0$) when we examined the effects of imperfect immunity conferred by transgenic *Wolbachia*. Finally, when calculating the trypanosomiasis transmission odds when a human or animal reservoir (or livestock) is

exposed, tsetse in compartments $\mathbf{V}_{pnI}$ and $\mathbf{V}_{ppI}$ need to be included in the total number of trypanosome-infected tsetse: equation (S35) was replaced with

$$V_I = \sum_a V_{nnIa} + V_{npIa} + V_{pnIa} + V_{ppIa}. \tag{S24}$$

## Cohabitation of multiple tsetse species

Although the absolute number of tsetse in an area stays relatively constant, different tsetse species within an area are known to fluctuate in numbers relative to each other over an annual period [9]. Areas inhabited by a single species of tsetse are generally characterized by recent environmental changes, such as deforestation or the elimination of wild hosts; cohabitation is much more common [9]. Additionally, interspecific mating among tsetse, if successful, generally produces hybrid offspring with limited or no fertility [10]. To examine the potential effects of releasing paratransgenic tsetse of a single species into an area cohabited by several species, and assuming that all tsetse have similar feeding and mating behaviors [9], we add a second tsetse species to our three-species SEIR model representing all other *Glossina* species not included in a paratransgenic release. In this scenario, only the fraction of all tsetse corresponding to the paratransgene tsetse release species can produce offspring with the paratransgene.

We extended the model (S10 & S34) to explicitly track this second species. Let $\mathbf{G}_S$, $\mathbf{G}_E$, $\mathbf{G}_I$ and $\mathbf{G}_R$ be age-structured vectors of tsetse of species not at risk for *Wolbachia* colonization that are trypanosomiasis susceptible, exposed, infected and non-susceptible, respectively, and

$$\mathbf{G} = \mathbf{G}_S + \mathbf{G}_E + \mathbf{G}_I + \mathbf{G}_R. \tag{S25}$$

The model equations for the non-target tsetse,

$$\frac{\mathrm{d}\mathbf{G}_S}{\mathrm{d}t} = \mathbf{B}_G\left[\mathbf{G} - \epsilon_V \mathbf{G}_I\right] + \mathbf{A}\mathbf{G}_S - \mathbf{D}_n\mathbf{G}_S - (a_H + a_L + v)\mathbf{G}_S, \tag{S26a}$$

$$\frac{\mathrm{d}\mathbf{G}_E}{\mathrm{d}t} = \mathbf{A}\mathbf{G}_E - \mathbf{D}_n\mathbf{G}_E - \tau_V\mathbf{G}_E + (a_H\lambda_{VH} + a_L\lambda_{VL})\mathbf{G}_S, \tag{S26b}$$

$$\frac{\mathrm{d}\mathbf{G}_I}{\mathrm{d}t} = \mathbf{A}\mathbf{G}_I - \mathbf{D}_n\mathbf{G}_I + \tau_V\mathbf{G}_E, \tag{S26c}$$

$$\frac{\mathrm{d}\mathbf{G}_R}{\mathrm{d}t} = \mathbf{A}\mathbf{G}_R - \mathbf{D}_n\mathbf{G}_R + \left[a_H(1-\lambda_{VH}) + a_L(1-\lambda_{VL}) + v\right]\mathbf{G}_S, \tag{S26d}$$

were added to system (S10 & S34). The birth matrix $\mathbf{B}_G$ corresponds to the fecundity matrix without any CI factors that was described previously,

$$\mathbf{B}_G = \begin{bmatrix} \leftarrow & Z_G(G)\mathbf{r} & \rightarrow \\ 0 & \dots & 0 \\ \vdots & & \vdots \\ 0 & \dots & 0 \end{bmatrix}, \tag{S27}$$

with

$$G = \sum_a G_a, \tag{S28}$$

and

$$Z_G(G) = 1 - \frac{G}{(1-\psi)K_V}, \tag{S29}$$

while the nonlinear term for births in targeted species (S15) was replaced by

$$Z(V) = 1 - \frac{V}{\psi K_V}. \tag{S30}$$

The parameter $\psi$ is approximately the proportion of the total tsetse population that are the targeted species. Finally, the number of infected tsetse that can transmit to vertebrate hosts includes all tsetse species, so equation (S35) was replaced with

$$V_I = \sum_a V_{nnIa} + V_{npIa} + G_{Ia}. \tag{S31}$$

## Remating

For remating, we distinguished between females with viable versus non-viable initial matings to determine the potential effects of CI inducing differential remating rates. The proportion of females with viable initial matings that remate is $\theta_c$ and the proportion of females with non-viable initial matings that remate is $\theta_i$. We assumed that all remating occurs in the next age class after initial mating, i.e. $i_R = 7$ (60–69 days after deposit as pupa). The matrix

$$\mathbf{R} = [r_{ij}], \tag{S32a}$$

with

$$r_{ij} = \begin{cases} \alpha & \text{if } i = i_R + 1,\ j = i_R, \\ 0 & \text{otherwise,} \end{cases} \tag{S32b}$$

which, analogous to $\mathbf{M}$, moves once-mated female tsetse from age class 7 to twice-mated females in age class 8. As before, a female mates with a random male from the population, of which the proportion $\phi$ are *Wolbachia* colonized, resulting in the terms $\pm(1-\phi)\theta_c\mathbf{R}\mathbf{V}_{pp}$ and $\mp\phi\theta_c\mathbf{R}\mathbf{V}_{pn}$ in (S10a & S10b), and their analogs in (S10c–S10j).

Tsetse can only become trypanosome-infected with *T. brucei* in their first blood mean and during the first twenty-four hours after emergence [4]. Thus the biting rates $a_H$ and $a_L$ on humans and animal reservoirs, and the rate $v = 1$ day$^{-1}$ moves tsetse that have not fed into the resistant classes. Infected tsetse enter exposed compartments and then progress to the infectious compartment at rate $\tau_V$.

For the vertebrate hosts, $H$ and $L$ denote humans and animal reservoirs, respectively. Their total population sizes are given by

$$H = H_S + H_E + H_I + H_R, \tag{S33a}$$

$$L = L_S + L_E + L_I + L_R, \tag{S33b}$$

and the model equations for humans and animal reservoirs follow standard SEIR vector-born pathogen models:

$$\frac{\mathrm{d}H_S}{\mathrm{d}t} = \delta_H H_R - a_H\beta_H V_I \frac{H_S}{H}, \tag{S34a}$$

$$\frac{\mathrm{d}H_E}{\mathrm{d}t} = a_H\beta_H V_I \frac{H_S}{H} - \tau_H H_E, \tag{S34b}$$

$$\frac{\mathrm{d}H_I}{\mathrm{d}t} = \tau_H H_E - \gamma_H H_I, \tag{S34c}$$

$$\frac{\mathrm{d}H_R}{\mathrm{d}t} = \gamma_H H_I - \delta_H H_R, \tag{S34d}$$

$$\frac{\mathrm{d}L_S}{\mathrm{d}t} = \delta_L L_R - a_L\beta_L V_I \frac{L_S}{L}, \tag{S34e}$$

$$\frac{\mathrm{d}L_E}{\mathrm{d}t} = a_L\beta_L V_I \frac{L_S}{L} - \tau_L L_E, \tag{S34f}$$

$$\frac{\mathrm{d}L_I}{\mathrm{d}t} = \tau_L L_E - \gamma_L L_I, \tag{S34g}$$

$$\frac{\mathrm{d}L_R}{\mathrm{d}t} = \gamma_L L_I - \delta_L L_R, \tag{S34h}$$

where

$$V_I = \sum_a V_{nnIa} + V_{npIa} \tag{S35}$$

is the number of trypanosome-infected tsetse summed over all age classes. All *T. brucei* transmission parameters are described in the main text.

# References

1. Alam U, Medlock J, Brelsfoard C, Pais R, Lohs C, et al. (2011) *Wolbachia* symbiont infections induce strong cytoplasmic incompatibility in the tsetse fly *Glossina morsitans*. PLoS Pathogens 7: e1002415.

2. World Health Organization (2013) Trypanosomiasis, Human African (sleeping sickness). Fact sheet 259, World Health Organization. URL `http://www.who.int/mediacentre/factsheets/fs259/en/`. Accessed on 11 January 2013.

3. Leak SGA (1999) Tsetse Biology and Ecology: Their Role in the Epidemiology and Control of Trypanosomosis. New York: CABI Publishing.

4. Rogers DJ (1988) A general model for the African trypanosomiases. Parasitology 97: 193-212.

5. Jarry M, Khaladi M, Gouteux JP (1996) A matrix model for studying tsetse fly populations. Entomologia Experimentalis et Applicata 78: 51-60.

6. Hu C, Rio RVM, Medlock J, Haines LR, Nayduch D, et al. (2008) The fitness cost of harboring an immunogenic parasite: tsetse–trypanosome interactions. PLoS Neglected Tropical Diseases 2: e192.

7. Jaenson TGT (1980) Mating behaviour of females of *Glossina pallidipes*. Bulletin of Entomological Research 70: 49-60.

8. Dame DA, Ford HR (1968) Multiple mating of *Glossina morsitans* Westw. and its potential effect on the sterile male technique. Bulletin of Entomological Research 58: 213-219.

9. Gouteux JP, Jarry M (1998) Tsetse flies, biodiversity and the control of sleeping sickness. Structure of a *Glossina* guild in southwest Côte d'Ivoire. Acta Oecologica 19: 453-471.

10. Gooding RH, Krafsur ES (2005) Tsetse genetics: Contributions to biology, systematics, and control of tsetse flies. Annual Review of Entomology 50: 101-123.
